# Mitochondrial matrix-localized Src kinase regulates mitochondrial morphology

**DOI:** 10.1007/s00018-022-04325-y

**Published:** 2022-05-30

**Authors:** Olivier Lurette, Hala Guedouari, Jordan L. Morris, Rebeca Martín-Jiménez, Julie-Pier Robichaud, Geneviève Hamel-Côté, Mehtab Khan, Nicholas Dauphinee, Nicolas Pichaud, Julien Prudent, Etienne Hebert-Chatelain

**Affiliations:** 1 Canada Research Chair in Mitochondrial Signaling and Physiopathology, Moncton, NB Canada; 2 Department of Biology, University of Moncton, Moncton, NB Canada; 3 Medical Research Council Mitochondrial Biology Unit, University of Cambridge, Cambridge Biomedical Campus, Cambridge, CB2 0XY UK; 4 Department of Chemistry and Biochemistry, University of Moncton, Moncton, NB Canada

**Keywords:** Mitochondrial dynamics, Cellular respiration, Mitochondria-shaping protein, Oxidative phosphorylation

## Abstract

The architecture of mitochondria adapts to physiological contexts: while mitochondrial fragmentation is usually associated to quality control and cell death, mitochondrial elongation often enhances cell survival during stress. Understanding how these events are regulated is important to elucidate how mitochondrial dynamics control cell fate. Here, we show that the tyrosine kinase Src regulates mitochondrial morphology. Deletion of Src increased mitochondrial size and reduced cellular respiration independently of mitochondrial mass, mitochondrial membrane potential or ATP levels. Re-expression of Src targeted to the mitochondrial matrix, but not of Src targeted to the plasma membrane, rescued mitochondrial morphology in a kinase activity-dependent manner. These findings highlight a novel function for Src in the control of mitochondrial dynamics.

**Supplementary Information:**

The online version contains supplementary material available at 10.1007/s00018-022-04325-y.

## Introduction

Mitochondria are considered as the cellular powerhouse due to their role in oxidative phosphorylation (OXPHOS) and ATP production, but they are also involved in other key physiological processes including Ca^2+^ homeostasis, apoptosis and steroidogenesis. Mitochondria form networks into the cytosol which are constantly modified by fission and fusion events, a process known as mitochondrial dynamics [1]. Morphology of individual organelle can shift from tubular to more fragmented or elongated structures according to the metabolic state of the cell, to respond to cellular cues and maintain cellular homeostasis [1, 2]. For instance, mitochondria elongate during mild stress [3, 4], whereas inhibition of OXPHOS or mitochondrial depolarization induces mitochondrial fragmentation [5, 6]. These morphological transition states are mainly regulated by dynamin GTPase proteins with Dynamin-related protein 1 (Drp1), the main actor of mitochondrial division, Mitofusins 1 and 2 (Mfn1/2) and Optic Atrophy 1 (OPA1) controlling outer mitochondrial membrane (OMM) and inner mitochondrial membrane (IMM) fusion, respectively [1, 7].

The tyrosine kinase Src was among the first oncogenes to be described [8]. It is involved in various physiological and oncogenic processes, ranging from metabolism, proliferation and differentiation to survival, motility and angiogenesis [8, 9]. Src has been found at multiple subcellular compartments including endosomes and plasma membrane [10], the Golgi apparatus [11, 12] as well as mitochondria [13, 14]. Src targets various mitochondrial proteins involved in different steps of metabolism including OXPHOS, fatty acid oxidation, pyruvate metabolism, ketone body production and production of reactive oxygen species [13, 15–17]. We recently observed that metastatic triple-negative breast cancer cells show higher activity of intramitochondrial Src [16]. Interestingly, metastatic properties of these cells depend on mitochondrial dynamics. Indeed, metastatic breast cancer cells display fragmented mitochondria, and inhibition of mitochondrial division via silencing of Drp1 or promotion of mitochondrial fusion by over-expression of Mfn1 blocks metastasis in these cells [18]. These findings suggest that Src could affect mitochondrial dynamics to control oncogenic properties, at least in specific cancer cells. However, the contribution of Src to adjustments of mitochondrial shape is currently not well understood.

The aim of this study was to examine and describe the potential role of Src in the regulation of mitochondrial dynamics. We observed that deletion or silencing of Src increased mitochondrial size, whereas its over-expression had the opposite effect. Deletion of Src also reduced cellular respiration and affected the levels of the mitochondria-shaping proteins Drp1, MiD51 and OPA1, whereas no effect was observed on mitochondrial mass, mitochondrial membrane potential and ATP levels. Re-expression of Src rescued alterations of mitochondrial morphology but not cellular respiration or levels of mitochondria-shaping proteins. Mechanistically, we propose that mitochondrial matrix-localized Src modulates mitochondrial shape independently of OXPHOS. Indeed, re-expression of Src targeted to the mitochondrial matrix rescued mitochondrial morphology, in a kinase activity-dependent manner, whereas re-expression of Src targeted to plasma membrane had no impact on mitochondrial shape. Overall, these findings highlight a direct contribution of intramitochondrial Src and its kinase activity to the control of mitochondrial dynamics.

## Results

### Src decreases mitochondrial size

**Fig. 1:**
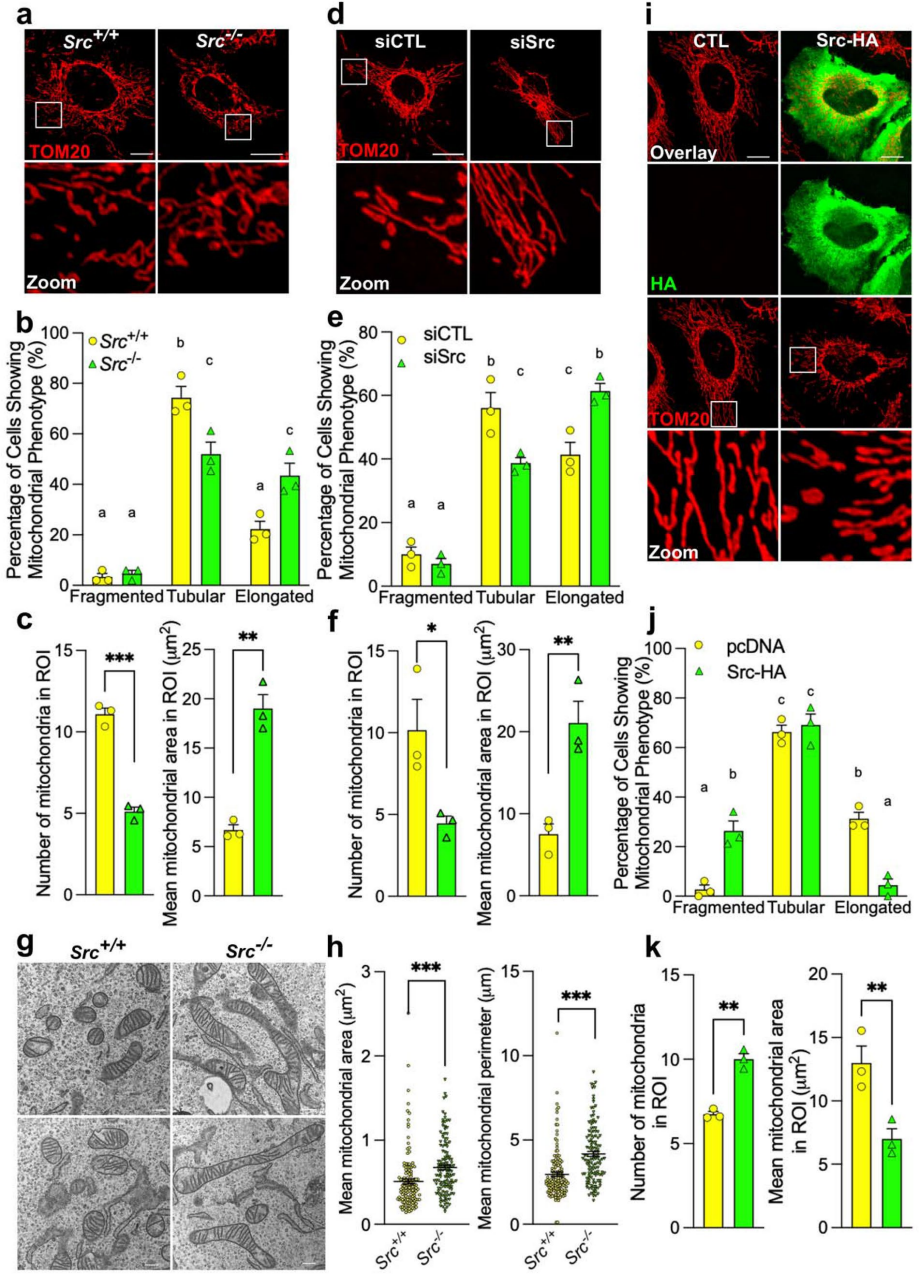
Src reduces mitochondrial size. **a** Representative micrographs (*n* = 3) of the mitochondrial protein TOM20 labeling in *Src*^+*/*+^ and *Src*^*−/−*^ mouse embryonic fibroblasts (MEFs). Scale bars: 20 μm. **b**, **c** Quantitative analysis of mitochondrial morphology showing **b** the distribution of cells among the different mitochondrial phenotypes, **c** the number of mitochondria and the area of individual mitochondria in regions of interest (ROI) in *Src*^+*/*+^ and *Src*^*−/−*^ MEFs as shown in **a**. **d** Representative micrographs (*n* = 3) of the mitochondrial protein TOM20 labeling in HeLa cells transfected with scramble siRNA (siCTL) or siRNA targeting *Src* (siSrc). Scale bars: 20 μm. **e**, **f** Quantitative analysis of mitochondrial morphology showing **e** the distribution of cells among the different mitochondrial phenotypes, **f** the number of mitochondria and the area of individual mitochondria in ROI in HeLa cells as shown in **d**. **g**, **h** Representative **g** transmission electron microscopy images of mitochondria and **h** quantification of mitochondrial area and perimeter in *Src*^+*/*+^ and *Src*^*−/−*^ MEFs. Scale bar: 500 nm. **i** Representative micrographs (*n* = 3) of the mitochondrial protein TOM20 and HA labeling in HeLa cells over-expressing control vector or Src-HA. **j**, **k** Quantitative analysis of mitochondrial morphology showing **j** the distribution of cells among the different mitochondrial phenotypes, **k** the number of mitochondria and the area of individual mitochondria in ROI in HeLa cells as shown in **i**. Data are presented as mean ± S.E.M. For the analyses of mitochondrial phenotype, data with different letters are statistically different (*p* < 0.05), according to two-way ANOVA followed by Tukey’s multiple comparison test. For all other analyses, *p < 0.05, **p < 0.01, ***p < 0.001 according to Student’s *t* test

Although indirect evidence suggests that Src might impact mitochondrial shape, its exact role in the regulation of mitochondrial dynamics remains unknown. To address this, the morphology of the mitochondrial network was first examined using confocal microscopy in (1) Src^++^ mouse embryonic fibroblasts (MEFs) expressing *Src* but knocked-out for *Yes* and *Fyn* (two other members of the Src kinases family) and (2) SYF MEFs knocked-out for *Src*, *Yes* and *Fyn* [19], named hereafter *Src*^+*/*+^ and *Src*^*−/−*^ MEFs, respectively. We observed that the deletion of Src (Fig. S1a) decreased the percentage of cells with tubular mitochondria and increased the number of cells with elongated mitochondria (Fig. 1a, b). Quantitative analyses of regions of interest (ROIs) showed that the deletion of Src also decreased the number of mitochondria and increased the area of individual organelles in ROIs (Fig. 1a, c), supporting the mitochondrial elongated phenotype observed in *Src*^−/−^ MEFs. To examine whether the downregulation of Src increases mitochondrial size independently of Yes and Fyn, Src was silenced using shRNA in naive MEFs (Fig. S1b). Similar to what was observed in *Src*^*−/−*^ MEFs, shSrc increased the percentage of MEFs with elongated mitochondria (Fig. S1c, d), decreased the number of individual mitochondria and increased the area of mitochondria in ROIs (Fig. S1c, e). Src was also silenced using siRNA in the human cell line HeLa (Fig. S1f). Again, we observed that lower expression of Src increased the percentage of HeLa cells with elongated mitochondria (Fig. 1d, e), decreased the number of individual mitochondria and increased the area of individual organelles in ROIs (Fig. 1d, f). The impact of Src deletion on mitochondrial morphology was confirmed using transmission electron microscopy since individual organelles of *Src*^−/−^ MEFs had higher area and perimeter as compared to *Src*^+/+^ mitochondria (Fig. 1g, h). Strikingly, upregulation of Src had opposite effects as compared to its deletion. Indeed, over-expression of Src-HA in Hela cells increased the percentage of cells with fragmented mitochondria (Fig. 1i, j) and increased the number of mitochondria with smaller area in ROIs (Fig. 1i, k). Overall, these findings reveal for the first time that Src reduces mitochondrial size.

**Fig. 2:**
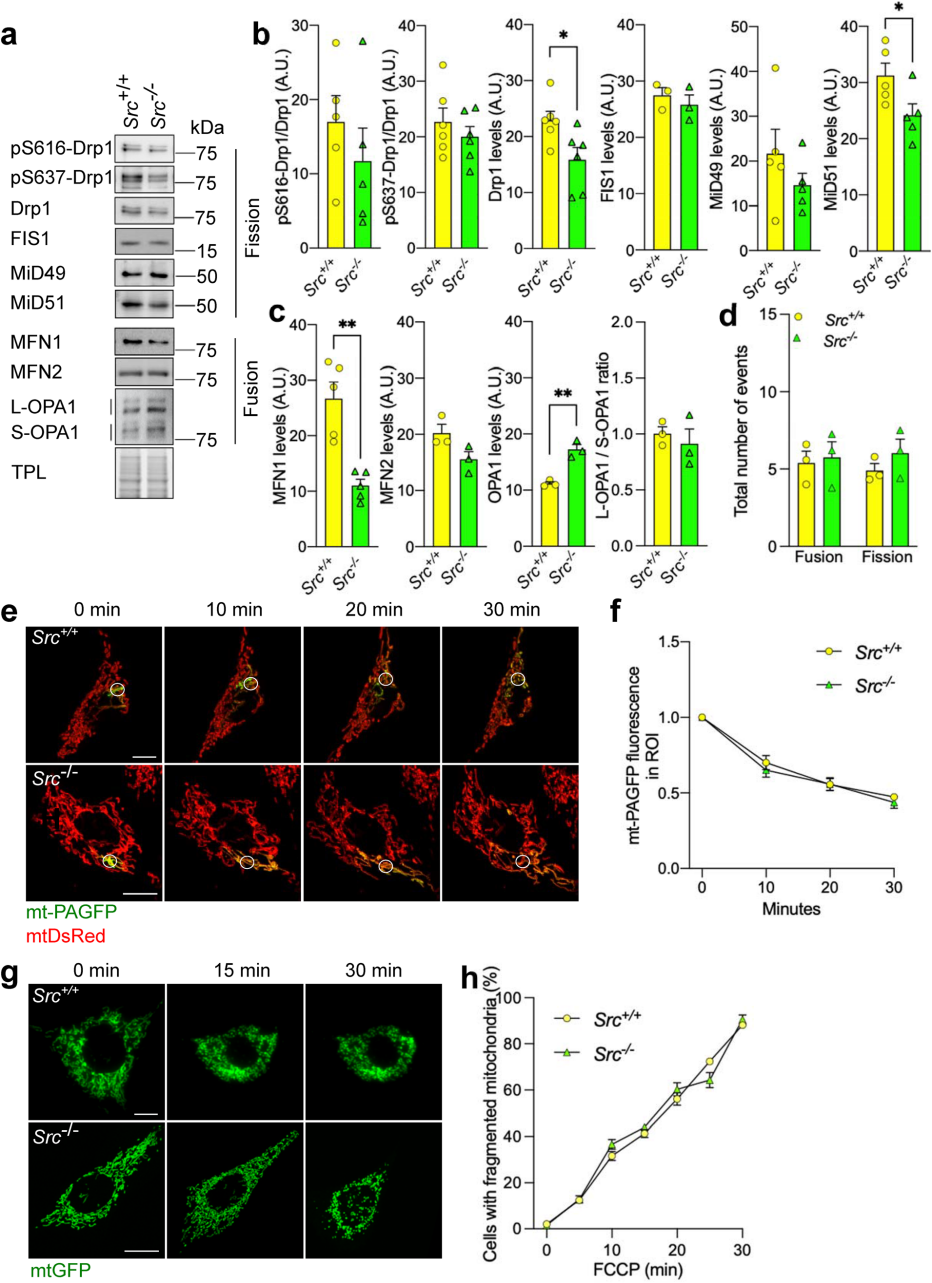
Src impacts on specific mitochondria-shaping proteins but not on mitochondrial fusion and fission rates. **a** Representative immunoblotting (*n* = 3–6) of the pro-fission proteins pS616-Drp1, pS637-Drp1, Drp1, FIS1, MiD49 and MiD51, of the pro-fusion proteins MFN1, MFN2 and OPA1, and of the total protein load (TPL) used as loading control, in *Src*^+*/*+^ and *Src*^*−/−*^ MEFs. **b**, **c** Quantification of the immunoblotting shown for **b** pro-fission and **c** pro-fusion mitochondria-shaping proteins. Protein levels were normalized by TPL. **d** Quantification of mitochondrial fusion and fission events in live *Src*^+*/*+^ and *Src*^*−/−*^ MEFs expressing mtGFP (*n* = 3) during 450 s videos. See also Movies 1 and 2. **e** Representative micrographs (*n* = 5) of *Src*^+*/*+^ and *Src*^*−/−*^ MEFs expressing the mitochondria-targeted photo-activable GFP (mt-PAGFP) and mtDsRed after photoactivation of mt-PAGFP. **f** Quantification of GFP normalized to DsRed in region of interest (ROI) upon photoactivation as shown in **e**. **g** Live cell micrographs of *Src*^+*/*+^ and *Src*^*−/−*^ MEFs expressing mtDsRed treated with FCCP (50 μM) as indicated. **h** Quantification of the number of cells with fragmented mitochondria as shown in **g**. Data are presented as mean ± S.E.M. A.U.: arbitrary unit. For **b**, **c** and **d**, *p < 0.05, **p < 0.01, according to Student’s *t* test. For **f** and **h**, data were analyzed with two-way ANOVA. Scale bars: 20 μm

To understand how the downregulation of Src leads to mitochondrial elongation, we first examined the levels of a panel of mitochondria-shaping proteins by immunoblotting (Fig. 2a–c). Results showed that levels of the pro-fission Drp1 and its mitochondrial receptor MiD51 were decreased in *Src*^−/−^ MEFs, whereas no change was observed in FIS1 or MiD49 levels (Fig. 2a, b). Levels of Drp1 were also lower in cytosolic and mitochondrial fractions of *Src*^−/−^ MEFs, but no differences were observed when normalized to the total level of Drp1 in TCL (Fig. S1h), suggesting no defects in Drp1 recruitment to mitochondrial membranes in *Src*^−/−^ MEFs. Activity and translocation of Drp1 to mitochondria are partly controlled by phosphorylation at its S616 and S637 residues [20–22]. When normalized to Drp1 levels, phosphorylation of both S616- and S637-Drp1 were not different between *Src*^+/+^ and *Src*^−/−^ MEFs (Fig. 2a, b), further corroborating that Src does not affect activity or subcellular distribution of Drp1. Among pro-fusion proteins, levels of the IMM fusion regulator OPA1 were increased in *Src*^−/−^ MEFs without changing the ratio between long and short OPA1 isoforms (Fig. 2a, c) or OPA1 oligomerization (Fig. S1i). The levels of the OMM fusion protein MFN2 did not change upon deletion of *Src* (Fig. 2a, c). Surprisingly, levels of the OMM fusion protein MFN1 were decreased in *Src*^−/−^ MEFs (Fig. 2a, c), suggesting it is not involved in Src-mediated regulation of mitochondrial morphology. These findings indicate that the mitochondrial elongation induced by deletion of Src is associated to lower levels of specific pro-fission proteins (Drp1 and MiD51) and higher levels of the pro-fusion protein OPA1.

### Src does not impact on mitochondrial fusion and fission rates

To examine whether the mitochondrial elongation induced by Src downregulation was associated with deregulation of mitochondrial fusion or fission, we first quantified real-time mitochondrial fusion and fission events in *Src*^+/+^ and *Src*^−/−^ MEFs expressing a GFP targeted to the mitochondrial matrix (mtGFP) by live cell confocal microscopy, as previously described [23]. Our findings suggest no difference in fusion and fission rates upon deletion of Src (Fig. 2d; Movies 1 and 2). Similar results were obtained when these events were quantified in HeLa cells treated with siSrc (Fig. S1g; Movies 3 and 4). Mitochondrial fusion was further evaluated using *Src*^+/+^ and *Src*^−/−^ MEFs expressing mitochondria-targeted photo-activable GFP (mt-PAGFP) [24]. Quantification of GFP fluorescence in stimulated ROIs of cells co-expressing mt-PAGFP and the mitochondrial marker mtDsRed indicated similar mitochondrial fusion rates between *Src*^+/+^ and *Src*^−/−^ MEFs after 10 min (Fig. 2e, f). We also treated *Src*^+/+^ and *Src*^−/−^ MEFs expressing mtGFP with the uncoupler carbonyl cyanide-*p*-trifluoromethoxyphenylhydrazone (FCCP) to induce Drp1-dependent mitochondrial fission [25]. We observed that *Src*^+/+^ and *Src*^−/−^ MEFs expressing mtGFP have similar fragmentation levels upon treatment with the uncoupler FCCP, suggesting no defects in the fission machinery per se, at least under this specific stress (Fig. 2g, h). These findings indicate that while we observed a dysregulation of some of the main regulators of mitochondrial morphology, including Drp1 and OPA1, the downregulation of Src increases mitochondrial size without affecting the overall mitochondrial fusion or fission rates.

**Fig. 3:**
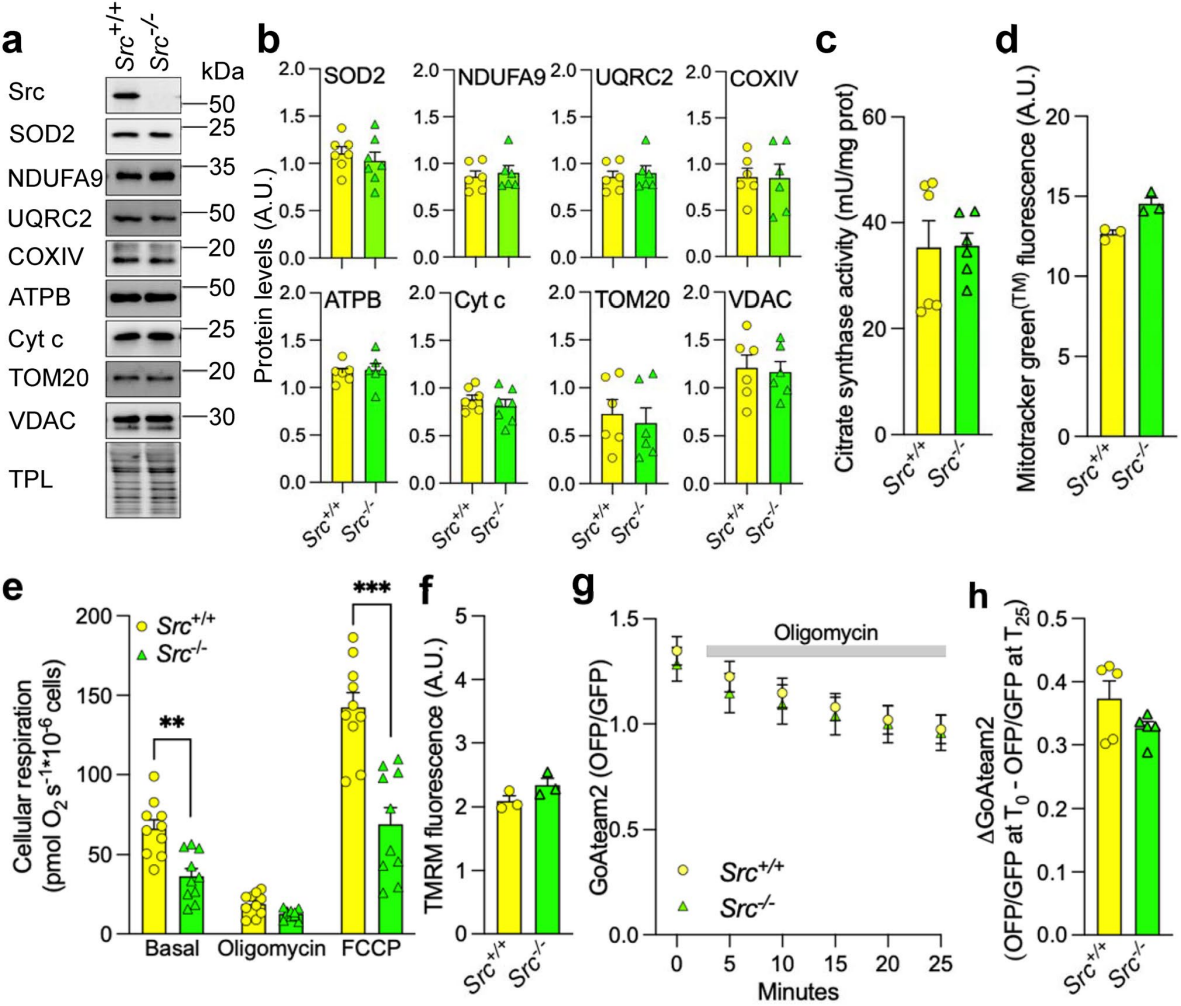
Silencing of Src does not affect mitochondrial mass, membrane potential and ATP levels. **a** Representative immunoblotting (*n* = 6–7) of mitochondrial proteins and of the total protein load (TPL) used as loading control in *Src*^+*/*+^ and *Src*^*−/−*^ MEFs. **b** Quantification of immunoblotting shown in **a**. Protein levels were normalized by TPL. **c** Citrate synthase activity in *Src*^+*/*+^ and *Src*^*−/−*^ MEFs. **d** Mitochondrial mass of *Src*^+*/*+^ and *Src*^*−/−*^ MEFs as measured by staining with Mitotracker Green™ (*n* = 3). **e** Cellular respiration of *Src*^+*/*+^ and *Src*^*−/−*^ MEFs (*n* = 10). **f** Mitochondrial membrane potential of *Src*^+*/*+^ and *Src*^*−/−*^ MEFs as measured using staining with TMRM (*n* = 3). **g** OFP/GFP ratio from the ATP FRET sensor GoAteam2 expressed in *Src*^+*/*+^ and *Src*^*−/−*^ MEFs before (time 0) and during treatment with oligomycin (1 μg mL^−1^) as indicated (*n* = 5). **h** Difference in OFP/GFP ratio from the ATP FRET sensor GoAteam2 expressed in *Src*^+*/*+^ and *Src*^*−/−*^ MEFs before (time 0) and after 25 min of treatment with oligomycin (1 μg mL^−1^) (*n* = 5), as shown in **e**. Data are presented as mean ± S.E.M. A.U.: arbitrary unit. Data were analyzed using Student’s *t* test (**b**–**d**, **f**, **h**) or two-way ANOVA followed by Tukey’s multiple comparison test (**e**, **g**). **p < 0.01, ***p < 0.001

Next, we observed that the changes of mitochondrial morphology induced by downregulation of Src were not associated with alterations of mitochondrial mass, as shown by immunoblotting several mitochondrial proteins (Fig. 3a, b), assessing citrate synthase activity (Fig. 3c), and by staining *Src*^+/+^ and *Src*^−/−^ MEFs with Mitotracker™ green which labels mitochondria independently of the mitochondrial membrane potential (Fig. 3d).

However, deletion of Src significantly decreased cellular respiration in MEFs (Fig. 3e). Similarly, silencing of Src in HeLa cells also decreased oxygen consumption (Fig. S1j), suggesting that the changes of mitochondrial size induced by Src downregulation could be due to defects in OXPHOS. However, staining *Src*^+/+^ and *Src*^−/−^ MEFs with tetramethylrhodamine (TMRM) showed that deletion of Src had no impact on mitochondrial membrane potential (Fig. 3f). Finally, cytosolic ATP levels, as measured by the ATP probe GoAteam2 [26], were not different between *Src*^+/+^ and *Src*^−/−^ MEFs (Fig. 3g). ATP levels were also comparable after treatment with the ATP synthase inhibitor oligomycin, used as a proxy to measure cytosolic ATP generated by the mitochondrial ATP synthase (Fig. 3g, h). Thus, these findings suggest that the Src-dependent decrease in oxygen consumption does not lead to altered mitochondrial membrane potential nor ATP production. Overall, we propose that the Src-dependent changes in mitochondrial size are not due to a global mitochondrial dysfunction.

### The kinase activity of mitochondrial matrix-localized Src is sufficient to impact on mitochondrial size

**Fig. 4:**
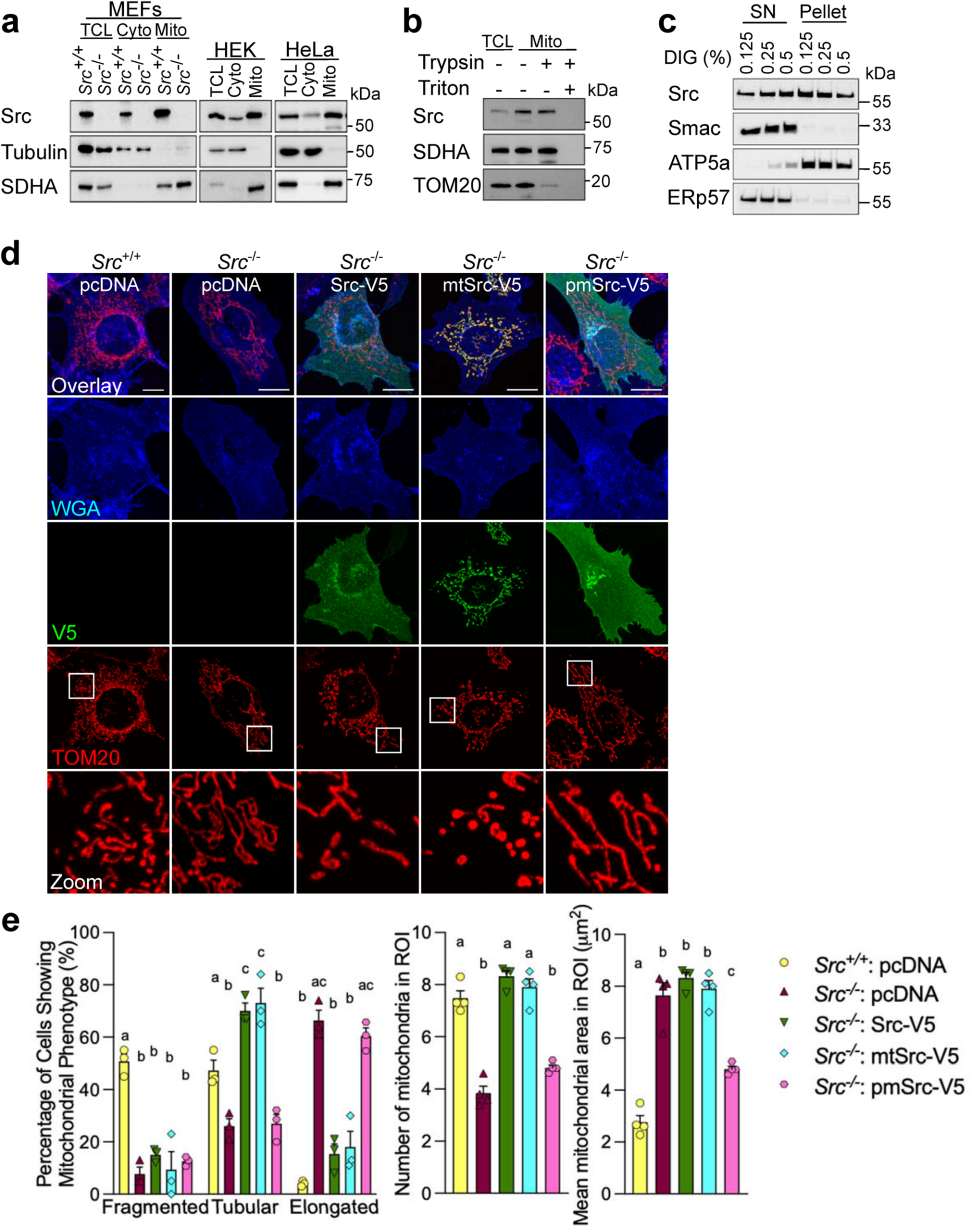
Mitochondrial matrix-localized Src regulates mitochondrial shape. **a** Representative immunoblotting (*n* = 3) of Src, cytosolic tubulin and mitochondrial SDHA in total cell lysate (TCL), cytosol- (cyto) and mitochondria- (mito) enriched fractions of *Src*^+*/*+^ and *Src*^*−/−*^ MEFs, HEK293 and HeLa. **b** Representative immunoblotting (*n* = 3) of Src, the IMM protein SDHA and the OMM protein TOM20 in mitochondria-enriched fractions of HeLa cells treated as indicated. **c** Representative immunoblotting (*n* = 4) of Src, Smac-diablo, ATP5a and ERp57 in supernatant (SN) and pellet obtained from mitochondria-enriched fractions of HeLa cells treated with digitonin as indicated. **d** Representative micrographs (*n* = 3) of the plasma membrane marker wheat germ agglutinin (WGA), V5 and TOM20 labeling in *Src*^+*/*+^ and *Src*^*−/−*^ mouse embryonic fibroblasts (MEFs) expressing pcDNA, Src-V5, mitochondria-targeted mtSrc-V5 and plasma membrane-targeted pmSrc-V5. **e** Quantitative analysis of mitochondrial morphology in region of interest (ROI) as shown in **c**. Scale bars: 20 μm. Data are presented as mean ± S.E.M. Data with different letters are statistically different (*p* < 0.05), according to one-way ANOVA followed by Tukey’s post hoc test

Src was previously shown to localize to different cellular compartments including mitochondria in several cellular and animal models, using multiple techniques such as transmission electron microscopy [13, 15, 27, 28]. Subcellular fractionation followed by immunoblotting showed that Src is present in mitochondria-enriched fractions obtained from MEFs, HEK293 and HeLa (Fig. 4a), as previously observed [13, 15, 16, 29]. Trypsin sensitivity assay then confirmed that Src is present inside mitochondria since it was protected from degradation when mitochondrial-enriched fractions were treated with trypsin, in contrast to the OMM protein TOM20 (Fig. 4b). To further determine the localization of Src at different mitochondrial subcompartments, mitochondria-enriched fractions were treated with increasing concentrations of digitonin, which dose-dependently release the intermembrane space protein Smac and the IMM matrix-facing protein ATP5a (Fig. 4c). We observed that the endogenous Src appears to be located in different mitochondrial compartments including the IMM/mitochondrial matrix compartment (Fig. 4b), as previously observed [13, 15]. Immunoblotting of the ER lumen protein ERp57 excluded bias linked to the well-known contamination of mitochondria-enriched fractions with ER since ERp57 was released differently than Src (Fig. 4c).

To test whether the impact of Src on mitochondrial morphology depends on its subcellular localization, we generated different tagged Src constructs: (i) untargeted Src, (ii) Src targeted to the mitochondrial matrix (mtSrc), and (iii) Src targeted to the plasma membrane (pmSrc). The constructs Src-V5, mtSrc-V5 and pmSrc-V5 were then used to rescue levels of Src in *Src*^−/−^ MEFs in specific subcellular compartments (Fig. 4d and Fig. S2a). Expression of Src-V5 or mtSrc-V5 both reversed the changes of mitochondrial shape induced by the deletion of Src, whereas no change was observed upon expression of pmSrc-V5 (Fig. 4d, e). Similarly, over-expression of Src-V5 and mtSrc-V5 in HeLa cells decreased mitochondrial size (Fig. S2b–d). Over-expression of Src targeted to the plasma membrane had no effect on the number and the area of individual mitochondria in ROIs although pmSrc-V5 changed the number of cells between the different mitochondrial phenotypes. (Fig. S2b–d). Globally, our results suggest that Src localized in the mitochondrial matrix is sufficient to affect mitochondrial shape.

**Fig. 5:**
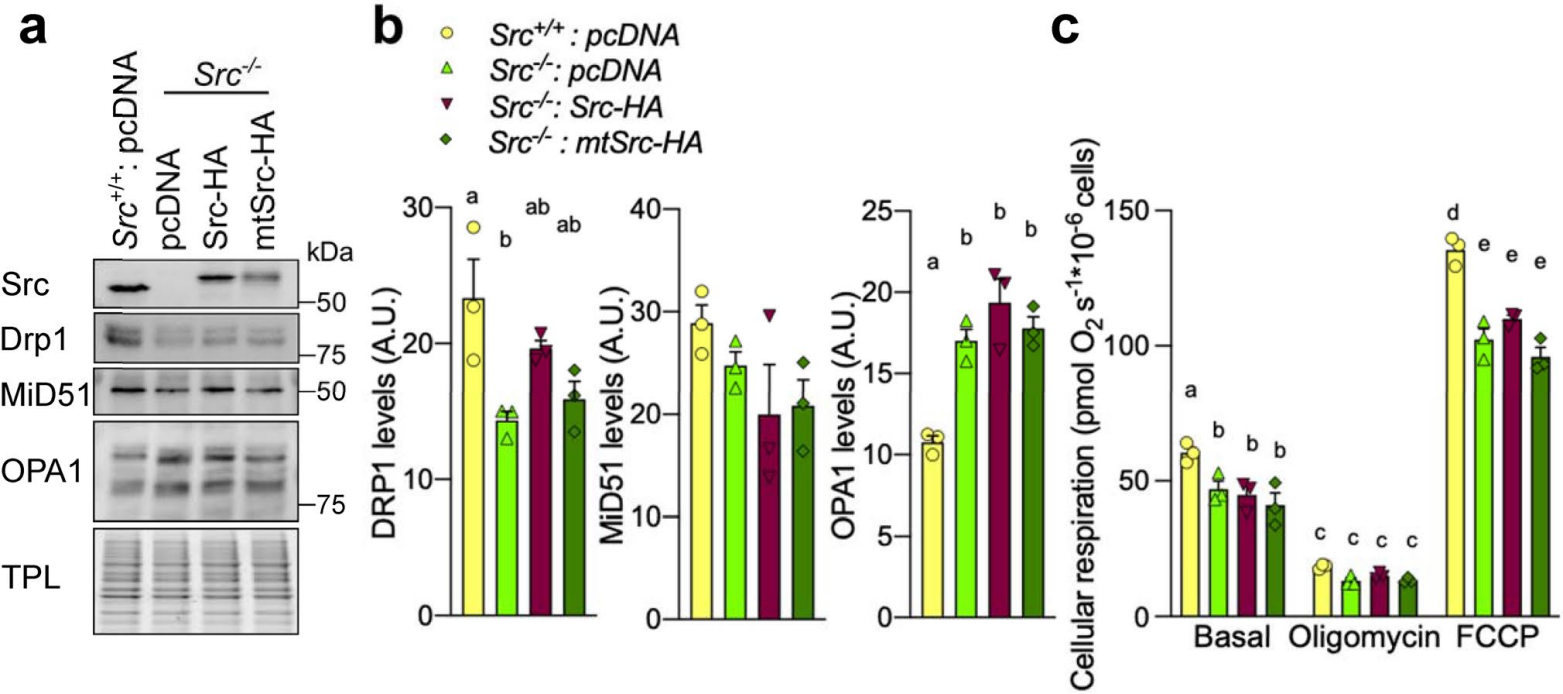
Mitochondrial matrix-localized Src modulates mitochondrial size independently of Drp1, MiD51, OPA1 and cellular respiration. **a** Representative immunoblotting (*n* = 3) of Src, Drp1, MiD51, OPA1 and total protein load (TPL), used as loading control, in *Src*^+*/*+^ and *Src*^*−/−*^ MEFs expressing pcDNA, Src-HA and mtSrc-HA. **b** Quantification of immunoblotting shown in **e**. Protein levels were normalized by TPL. **c** Cellular respiration of *Src*^+*/*+^ and *Src*^*−/−*^ MEFs expressing pcDNA, Src-HA and mtSrc-HA (*n* = 3). Data with different letters are statistically different (*p* < 0.05), according to one-way ANOVA (for **b**) or two-way ANOVA (for **c**) followed by Tukey’s post hoc test. *A.U.* arbitrary unit

Since re-expression of Src in *Src*^−/−^ MEFs rescued mitochondrial morphology, we tested whether it would also reverse the alterations of Drp1, MiD51 and OPA1 levels. Surprisingly, expression of Src-HA and mtSrc-HA in *Src*^−/−^ MEFs did not reverse the decrease of Drp1 and MiD51 levels or the increase of OPA1 levels induced by the deletion of *Src* (Fig. 5a, b). Considering that downregulation of Src decreases cellular respiration (Fig. 3e and Fig. S1j), we also examined whether expression of Src-HA and mtSrc-HA would affect oxygen consumption in *Src*^−/−^ MEFs. Similar to Drp1, MiD51 and OPA1 levels, expression of Src-HA and mtSrc-HA in *Src*^−/−^ MEFs did not rescue cellular respiration (Fig. 5c). Altogether, these results suggest that Src affects mitochondrial size and cellular respiration via different mechanisms.

**Fig. 6:**
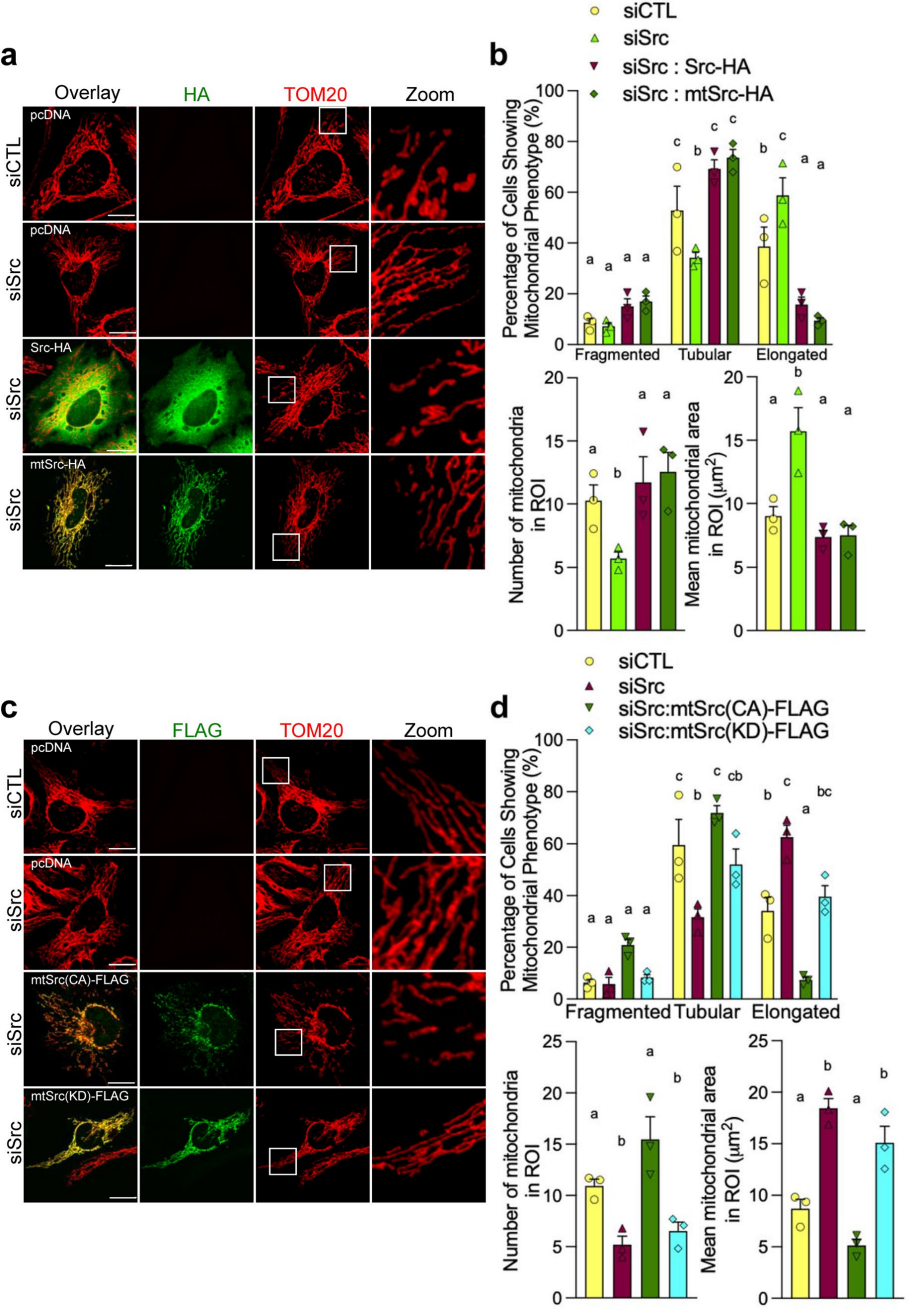
The kinase activity of mitochondrial matrix-localized Src affects mitochondrial shape. **a** Representative micrographs (*n* = 3) of TOM20 and HA labeling in HeLa cells transfected with scramble siRNA (siCTL) or siRNA targeting *Src* (siSrc) with pcDNA, Src-HA or mitochondria-targeted mtSrc-HA. **b** Quantitative analysis of mitochondrial morphology showing the distribution of cells among the different mitochondrial phenotypes, the number of mitochondria and the area of individual mitochondria in region of interest (ROI) in HeLa cells as shown in **a**. **c** Representative micrographs (*n* = 3) of TOM20 and FLAG labeling in HeLa cells transfected with scramble siRNA (siCTL) or siRNA targeting *Src* (siSrc) and over-expressing pcDNA, mitochondria-targeted constitutively active mtSrc(CA)-FLAG or mitochondria-targeted kinase-dead mtSrc(KD)-FLAG. **d** Quantitative analysis of mitochondrial morphology showing the distribution of cells among the different mitochondrial phenotypes, the number of mitochondria and the area of individual mitochondria in ROI in HeLa cells as shown in **c**. Data are presented as mean ± S.E.M. Data with different letters are statistically different (*p* < 0.05), according to one-way ANOVA followed by Tukey’s post hoc test. *Scale bars: 20 μm*

Expression of Src-HA and mtSrc-HA both reversed the mitochondrial elongation phenotype induced by siSrc in HeLa cells (Fig. 6a, b). To examine if these morphological changes depend on the kinase activity of Src, we performed similar rescue experiments in HeLa cells treated with siSrc using constitutively active (CA) and kinase-dead (KD) mutants of mtSrc [30], named mtSrc(CA)-FLAG and mtSrc(KD)-FLAG, respectively. Interestingly, mtSrc(CA)-FLAG, but not the inactive mutant mtSrc(KD)-FLAG, reversed the mitochondrial elongation induced by siSrc in HeLa cells (Fig. 6c, d), suggesting that mitochondrial matrix-localized Src affects mitochondrial shape in a kinase activity-dependent manner. The same constructs were then over-expressed in HeLa cells: mtSrc(CA)-FLAG reduced mitochondrial size, whereas the inactive mutant mtSrc-(KD)-FLAG had no effect (Fig. S3a, b). This gain-of-function experiment confirmed that the kinase activity of Src inside the mitochondrial matrix is sufficient to induce the Src-dependent alterations of mitochondrial shape.

### Over-expression of mitochondrial matrix-localized Src prevents mitochondrial elongation during starvation

**Fig. 7:**
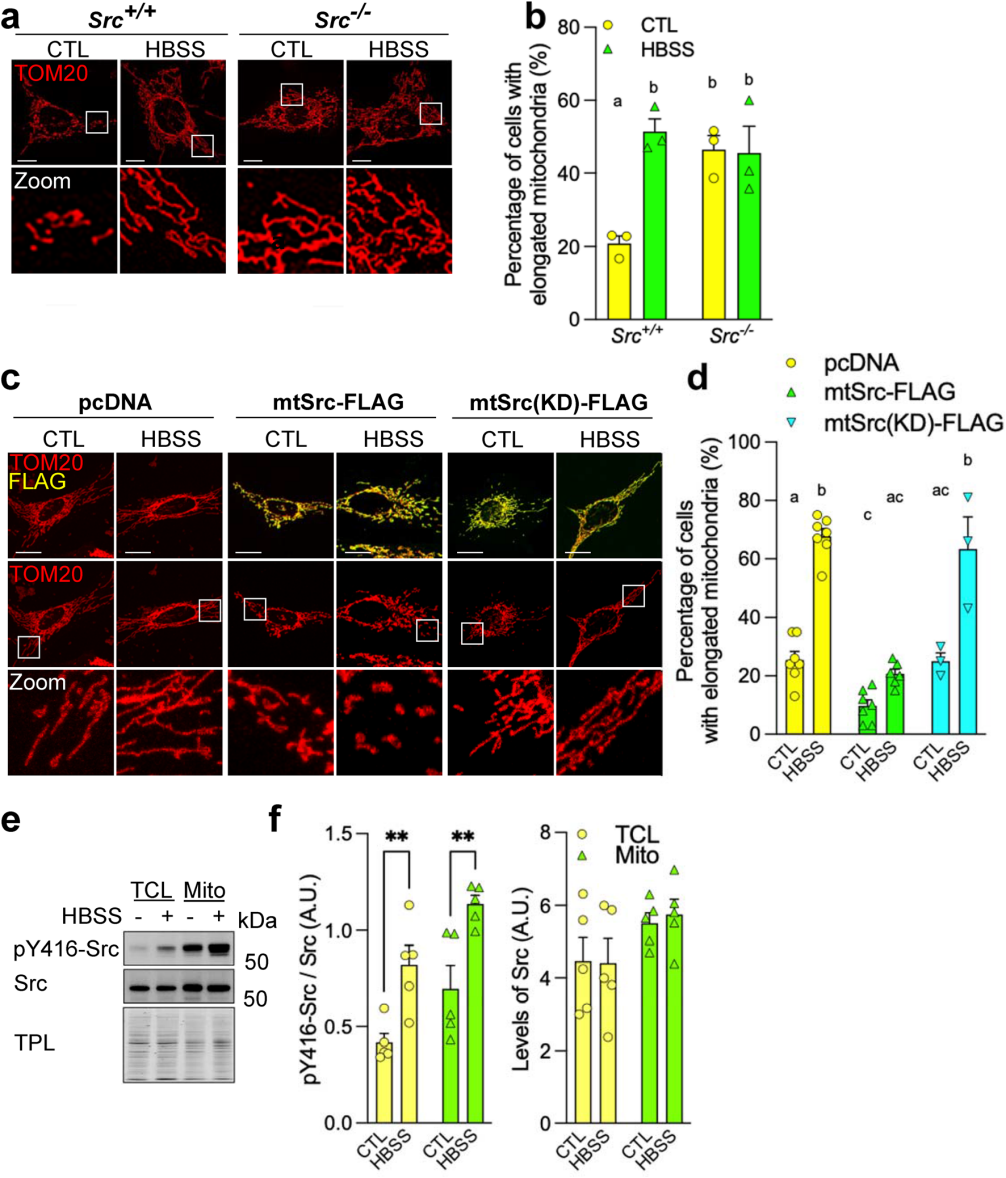
Endogenous mitochondrial matrix-localized Src does not modulate starvation-induced mitochondrial elongation. **a** Representative micrographs of TOM20 labeled in *Src*^+*/*+^ and *Src*^*−/−*^ MEFs treated with HBSS during 5 h (*n* = 3). **b** Quantitative analysis of mitochondrial morphology showing the percentage of cells with elongated mitochondria, as shown in **a**. **c** Representative micrographs of TOM20 and FLAG labeled in HeLa cells over-expressing mitochondria-targeted mtSrc-FLAG, or mitochondria-targeted kinase-dead mtSrc(KD)-FLAG, and treated with HBSS (5 h) as indicated (*n* = 3–6). **d** Quantification of the number of cells with elongated mitochondria as shown in **c**. **e** Representative immunoblotting (*n* = 3) of pY416-Src, Src and total protein load (TPL) in total cell lysates (TCL) and mitochondria-enriched fractions (Mito) obtained from HeLa cells treated with HBSS during 5 h. **f** Quantification of the activity (left) and levels (right) of Src based on the immunoblotting shown in **e**. Protein levels were normalized by TPL. Data are shown as mean ± S.E.M. A.U.: arbitrary unit. Data with different letters are statistically different (*p* < 0.05), according to two-way ANOVA followed by Tukey’s post hoc test (for **b** and **d**). **p < 0.01 according to Student’s *t* test. Scale bars: 20 μm

During starvation, mitochondria elongate to protect themselves from autophagic degradation and prevent cell death [4, 31]. We thus hypothesized that the Src-dependent modulation of mitochondrial dynamics could be involved in this process. To address this, we first treated *Src*^+/+^ and *Src*^−/−^ MEFs with Hank’s balanced salt solution (HBSS) during 5 h, a treatment known to induce starvation-induced mitochondrial elongation [4]. We observed more *Src*^+/+^ MEFs with elongated mitochondria upon starvation, whereas no change was observed in *Src*^−/−^ MEFs (Fig. 7a, b), confirming that the mitochondrial network of *Src*^−/−^ MEFs was already in an elongated state before starvation. The mitochondrial morphology was also examined in HeLa cells over-expressing different Src constructs and treated with HBSS during 5 h. As expected, the HBSS treatment induced mitochondrial elongation in control HeLa cells (Fig. 7c, d). Mitochondrial elongation induced by starvation was, however, inhibited in cells over-expressing mtSrc-FLAG but not in cells expressing the inactive mtSrc(KD)-FLAG (Fig. 7c, d), suggesting that the activity of intramitochondrial Src is decreased during starvation to allow mitochondrial elongation. However, we observed higher phosphorylation of the activating residue of Src (i.e., pY416-Src), indicating that Src activity increased upon HBSS treatment in both total cell lysates and mitochondria-enriched fractions (Fig. 7e, f). This finding indicates that the regulation of mitochondrial shape by endogenous mitochondrial matrix-localized Src is likely not involved in starvation-induced mitochondrial elongation.

## Discussion

The present work examined the impact of the tyrosine kinase Src on mitochondrial morphology. We show that lower expression of Src increases mitochondrial size, whereas its over-expression shortens mitochondria. We suggest that the kinase activity of Src inside the mitochondrial matrix adjusts mitochondrial shape.

Mitochondrial activity and morphology are tightly connected and treatment with inhibitors of OXPHOS leads to mitochondrial fragmentation [5, 6]. For instance, the uncoupler FCCP or the mitochondrial ATP synthase inhibitor oligomycin induce mitochondrial fragmentation via the cleavage of OPA1 by the protease OMA1, leading to inhibition of mitochondrial fusion and unopposed mitochondrial fragmentation [32]. Previous works showed that pharmacological inhibition or genetic downregulation of Src decreases OXPHOS, mitochondrial membrane potential and ATP production [13, 15, 33, 34]. Here, the downregulation of Src did not decrease mitochondrial membrane potential or ATP levels, at least in the cytosol, suggesting that the alterations of oxygen consumption induced by downregulation of Src observed here and in previous work [13, 15, 33–35] did not translate into decreased mitochondrial membrane potential or ATP levels in MEFs and HeLa cells. Our results thus suggest that the Src-dependent regulation of mitochondrial morphology is independent of OXPHOS defects. In contrast, mitochondria can elongate upon various stress stimuli. Starvation triggers cAMP-PKA signaling and inhibition of Drp1-mediated mitochondrial fragmentation leading to unopposed mitochondrial fusion [4, 31]. UV irradiation, actinomycin D and cycloheximide also induce mitochondrial elongation. In such conditions, mitochondrial fusion increases due to stabilization of the pro-fusion long OPA1 isoforms by SLP-2 [3]. This stress-induced mitochondrial hyperfusion is independent of mitochondrial depolarization or changes in ATP levels [3, 4], similar to our observations about Src-dependent mitochondrial elongation. However, our findings suggest that the regulation of mitochondrial size by endogenous Src is not involved in starvation-induced mitochondrial elongation. Overall, the functional impact of the Src-mediated modulation of mitochondrial shape is still unclear, and future works will be necessary to address this.

Our findings suggest that Src regulates mitochondrial shape downstream of mitochondria-shaping proteins. Indeed, although levels of Drp1, MiD51 and OPA1 were different between *Src*^+/+^ and *Src*^−/−^ MEFs, re-expression of Src in *Src*^−/−^ MEFs rescued the changes of mitochondrial morphology but not of the levels of these mitochondria-shaping proteins. Thus, the different levels of Drp1, MiD51 and OPA1 between *Src*^+/+^ and *Src*^−/−^ MEFs could have been indirect consequence of the deletion of the kinase itself, to compensate for the chronic deficiency of Src. Similarly, deletion of Src did not affect processing and oligomerization of OPA1. Thus, Src could control mitochondrial shape downstream of the canonical fission/fusion machineries. Drp1-independent mitochondrial division can occur during hypoxia or deferiprone-induced mitophagy in mammalian cells [36]. Elevated cytosolic Ca^2+^ can also shorten mitochondria independently of Drp1 [37, 38]. Moreover, some types of mitochondria-derived vesicles are released from mitochondria in a Drp1-independent manner [39]. To our knowledge, there is, however, no evidence for any mechanism leading to mitochondrial elongation independently of the pro-fusion proteins OPA1 and Mfn1/2. Although this was not the focus of this work, it would be critical to identify the mechanisms linking Src and mitochondrial dynamics in future studies.

Src is present in several subcellular compartments, including mitochondria [10, 13, 15]. Our experiments confirm previous observations showing that a pool of Src is, at least partly, localized inside the organelle [13, 15, 28]. Targeting Src to the mitochondrial matrix was sufficient to reduce mitochondrial size. These findings suggest that Src could target proteins inside the organelle to control its shape. We recently identified several potential targets of intramitochondrial Src which could be involved in the morphological changes observed in the present work [17]. For example, it has been shown that Src could interact with SLP-2 [17] which is required for stress-induced mitochondrial hyperfusion [3]. Similarly, we showed potential interaction of intramitochondrial Src with prohibitin 2 and the mitochondrial contact site and cristae organizing system Mic60 [17] which are also key players in the regulation of mitochondrial architecture [40, 41]. It is also possible that Src is present in other compartments of the organelle from where it could impact on mitochondrial architecture and functions. Similarly, over-expression of Src targeted to the plasma membrane impacted the number of cells among the different mitochondrial phenotypes, suggesting that the different subcellular pools of Src could impact on mitochondrial morphology via specific pathways. Further investigation could thus allow to identify new pathways important for the regulation of mitochondrial shape and examine whether Src modulates mitochondrial morphology and functions through SLP-2, Mic60 and other classical and non-classical mitochondria-shaping proteins.

In conclusion, the present work highlights a novel function for Src as a regulator of mitochondrial morphology. This may provide insights into the contribution of Src signaling in several cellular processes and diseases such as the involvement of Src in different pathologies, including cancer. The role of Src in oncogenesis and the metabolic rewiring observed in cancer cells is well described [8]. Considering that metastasis of some cancer cells depends on appropriate regulation of mitochondrial dynamics, the intramitochondrial Src signaling and its impact on mitochondrial morphology could represent a novel therapeutic avenue.

## Materials and methods

### Cell culture and transfection

Src^++^ and SYF MEFs [19] (named here *Src*^+*/*+^ and *Src*^*−/−*^, respectively), naive MEFs, HeLa and HEK293 cells were cultured in high glucose (4.5 g L^−1^) Dulbecco’s modified Eagle’s medium (DMEM) supplemented with 2 mM glutamine, 1 mM pyruvate, 10% (v/v) of fetal bovine serum (FBS), 100 units mL^−1^ penicillin and 100 g mL^−1^ streptomycin. Cells were cultured at 37 °C in 5% CO_2_ and 95% humidity. HeLa and HEK293 cells were transiently transfected with polyethylenimine (PolySciences, PA, USA), FuGENE HD (Promega, WI, USA) or Lipofectamine™ RNAiMAX (Invitrogen, CA, USA), whereas MEFs were electroporated with the Neon™ transfection system (Invitrogen, CA, USA) in accordance with the manufacturer’s instructions. Cells were analyzed 24–48 h following transfection.

### Constructs and site-directed mutagenesis

The constructs encoding hemagglutinin (HA)- or V5-tagged mouse Src (Src-HA and Src-V5) and HA- or V5-tagged mouse Src targeted to the mitochondria (mtSrc-HA and mtSrc-V5) were generated by amplification of the sequence of *Src* from pCMV5-Src (#13663, Addgene, MA, USA) and fused to HA or V5 with or without the mitochondrial leading sequence (MLS) of cytochrome *c* oxidase VIIIa, as described [16]. Constructs encoding FLAG-tagged human wild-type Src, constitutively active (CA) Src and kinase-dead (KD) Src fused to the MLS of the very long chain acyl-CoA dehydrogenase (named hereafter mtSrc-FLAG, mtSrc(CA)-FLAG and mtSrc(KD)-Flag, respectively) were obtained from Addgene (#44652, #44654 and # 44653, respectively). Src-V5 was targeted to the plasma membrane (pmSrc-V5) using the first 26 amino acids of Lck (derived from Lck-GFP obtained from Addgene #61099) as previously shown [42, 43].

### RNAi

Designed individual or SMARTpool siRNAs were used to specifically knockdown Src in HeLa cells. Single siRNA was purchased from Sigma-Aldrich (MO, USA) and were based on the following sequences: (sense: 5′-GGAAACACCUGUAGGCAGAUU-3′; antisense: 5′-UCUGCCUACAGGUGUUUCCUU-3′). SMARTpool siRNAs were obtained from Dharmacon (L-003175-00-0005 ON-TARGETplus Human SRC siRNA). Scrambled siRNA (siCTL) was obtained from ThermoFisher Scientific (#4390843). HeLa cells were transfected with siRNA (20 nM) using Lipofectamine™ RNAiMax (Invitrogen, CA, USA) according to the manufacturer’s protocol. For rescue experiments, cells were transfected with plasmids encoding empty vector or Src constructs 48 h after transfection with siSrc. Cells were then analyzed 24 h later.

Control shRNA (shCTL, #SCH016) and shRNA against mouse Src (shSrc, # TRCN0000278660) were purchased from Sigma-Aldrich. Naive MEFs were electroporated with mtGFP and either shCTL or shSrc, as described above. MEFs were analyzed 24 h later.

### Subcellular fractionation

Mitochondria-enriched fractions were obtained as previously described [44]. Briefly, cells were harvested and resuspended in mitochondrial isolation buffer (250 mM sucrose, 1 mM EDTA, 5 mM HEPES, pH 7.4) supplemented with 1% protease inhibitor cocktail and 2 mM sodium orthovanadate. Cells were lysed with 15 strokes using a 25-gauge syringe on ice and centrifuged at 1500*g* for 5 min (4 °C). The resulting supernatant (TCL) was centrifuged at 12,500*g* for 10 min (4 °C). The obtained supernatant was considered as the cytosol-enriched fraction, whereas the pellet was resuspended in the mitochondrial buffer and a cycle of centrifugations at 1500*g* and 12,500*g* was repeated. The final pellet was considered as the mitochondria-enriched fraction.

For assessing distribution of Src among mitochondrial subcompartments, mitochondria-enriched fractions were resuspended in 1 M HEPES–KOH buffer (pH 7.4) in the presence of 0.125, 0.25 or 0.5% of digitonin (Sigma-Aldrich, MO, USA) and incubated at room temperature (RT) with continuous shaking at 1000 rpm. After 15 min, samples were centrifuged at 12,500*g* at 4 °C for 10 min. The resulting pellets and supernatants were processed for SDS-PAGE. The presence of proteins from different mitochondrial subcompartments in pellets and supernatants was analyzed using immunoblotting.

### Fluorescence microscopy

For the evaluation of mitochondrial membrane potential and mitochondrial mass, *Src*^+/+^ and *Src*^−/−^ MEFs were treated with 200 nM tetramethylrhodamine methyl ester (TMRM, Invitrogen, CA, USA) and with 100 nM Mitotracker green™ (Invitrogen, CA, USA), respectively, during 30 min at 37 °C. Cells were then rinsed and directly imaged with the EVOS Fl Auto 2 (Invitrogen, CA, USA) at 37 °C in 5% CO_2_ and 95% humidity using a 40 × objective (LPLAN 40 ×, 0.65NA, EVOS), and appropriate excitation and emission filters. For each independent experiment, fluorescence intensity was quantified in 30 cells using Fiji software (NIH, MD, USA).

For analysis of mitochondrial morphology, cells seeded on 12- or 18-mm glass round coverslips were fixed in 4–5% (w/v) paraformaldehyde for 15 min at RT and then incubated with 50 mM ammonium chloride in PBS for 10 min at RT. Cells were permeabilized with 0.1–0.5% triton X-100/PBS for 10–15 min and blocked in 10% BSA or FBS in PBS for 45 min, as previously described [46]. Cells were labeled between 1 h and overnight with rabbit anti-HA (Cell Signaling; #2367), rabbit anti-TOM20 (Proteintech 11802-1-AP), rabbit anti-myc (Cell Signaling: #2278), mouse anti-HA (Biolegend; #901513), mouse anti-myc (Cell Signaling; #2276), mouse anti-FLAG (Sigma-Aldrich; #F1804) and mouse anti-TOM20 (Santa Cruz; sc-17764) antibodies, and then with anti-rabbit IgG or anti-mouse IgG conjugated with Alexa Fluor^®^488, Alexa Fluor^®^546, Alexa Fluor^®^633 or Alexa Fluor^®^647 (Invitrogen, CA, USA) for 1 h at RT in either PBS-BSA or PBS-FBS. Plasma membrane was labeled with wheat germ agglutinin conjugated with Alexa Fluor^®^647 (Invitrogen, CA, USA). For Figs. 1a, d, i, 7a, c, Figs. S1c, S2c images were acquired using a 60 × or 100 ×/1.4 apochromat objective of the Nikon Eclipse Ti-E microscope, coupled to an Andor Dragonfly spinning disk confocal system equipped with an Andor sCMOS camera, exciting with appropriate lasers. Fusion software (Andor) was used to acquire images. For Figs. 2e, g, 4d, 6a, c and Fig. S3a, coverslips were mounted on slides and placed on the stage of the Olympus FV3000 confocal microscope (Olympus, Japan) and imaged using a 60 × oil objective (UPLAN 60 × oil, 1.35NA, Olympus, Japan), and appropriate excitation/emission parameters. Stacks separated by 0.2 μm along the *z* axis were acquired. Images were then complied by “max projection” using Fiji software.

For analysis of the mitochondrial morphology, cells were first presented as fragmented, tubular and elongated. The mitochondrial network was classified as fragmented when mitochondria are short and spherical; elongated when > 50% of mitochondria are longer than 5 μm and highly interconnected; tubular when the mitochondrial network appeared as an intermediate between fragmented and elongated. At least 30 cells were analyzed in each independent experiment (*n* = 3) using Fiji software.

For analysis of the mitochondrial number and individual mitochondrial area, ROIs of 225 μm^2^ were randomly selected within peripheral regions of cells from max projection images and followed by manual thresholding, as previously described [45]. Mitochondrial number and size in ROI were obtained using the Analyze particles plugin in Fiji with a minimum area of 0.2 μm^2^. At least 10 cells were analyzed for each independent experiment (*n* = 3, minimum of 30 cells in total per condition).

The number of mitochondrial fusion and fission events in live cells was analyzed as previously described [23]. Briefly, cells expressing mtGFP were imaged every 2.5 s for 450 s, and then analyzed using Fiji in at least 5 cells in 3–5 independent experiments (total of 15 cells for MEFs and a total of 25 cells for HeLa). Fusion and fission events were calculated in ROI of 225 μm^2^ randomly selected within peripheral regions of cells from max projection images. A single fission event was noted when one mitochondrion divided in two and remained separated for at least the next two timeframes. A fusion event was noted when two adjacent organelles connected and remained fused for at least the next three timeframes.

Mitochondrial fusion was also analyzed using live cell imaging of *Src*^+/+^ and *Src*^−/−^ MEFs co-expressing mtDsRed and photo-activable mitochondria-targeted GFP (PA-mtGFP, Addgene, #23348). PA-mtGFP was photoactivated with 405 nm laser (at 2% laser intensity) during 1 s in a randomly selected ROI of 10 μm^2^. Cells were then imaged every 10 min using a 60 × oil objective (UPLAN 60 × oil, 1.35NA, Olympus). GFP and DsRed fluorescence in ROIs were analyzed using Fiji in 5–8 cells in 5 independent experiments (a total of 37 cells per condition). GFP fluorescence was normalized by DsRed fluorescence.

Mitochondrial fission was also analyzed using live cell imaging of *Src*^+/+^ and *Src*^−/−^ MEFs expressing mtGFP and treated with 10 µM carbonyl cyanide-*p*-trifluoromethoxyphenylhydrazone (FCCP) at 37 °C in 5% CO_2_ and 95% humidity. Live cells were examined at different time points using the EVOS FL Auto2 imaging system and a 40 × objective (LPLAN 40 ×, 0.65NA, EVOS), with appropriate excitation and emission filters. At least 40 cells were analyzed for each independent experiment (*n* = 4).

ATP levels were evaluated using the ATP FRET sensor GoAteam2, as described [26]. *Src*^+/+^ and *Src*^−/−^ MEFs expressing GoAteam2 were analyzed 24 h post-transfection, with excitation at 488 nm and emission at 500–535 nm for GFP and 545–625 nm for OFP. To determine ATP levels depending on the mitochondrial ATP synthase, MEFs were treated with oligomycin (1 µg/ml). The OFP/GFP ratio was measured every 5 min in a randomly selected ROI of 25 μm^2^ within peripheral regions of cells using Fiji in a minimum of 5 cells in 5 independent experiments (total of 25 cells per condition).

Several cells were analyzed in each independent experiment (as indicated). To minimize bias and make sure the analyzed cells represented the overall population of cells, coverslips were divided in four quarters for each independent experiment, from which an approximate equal number of cells were randomly selected, When appropriate, ROIs were selected within peripheral regions to ensure an appropriate distribution of all organelles, as described previously [45], in contrast to the perinuclear region, which is known to “compact” organelles due to steric hindrance leading to changes in their morphologies.

### OPA1 oligomerization

*Src*^+/+^ and *Src*^−/−^ MEFs were treated with the crosslinker bismaleimidohexane (1 mM) during 30 min at 37 °C. Upon treatment, MEFs were directly processed for SDS-PAGE.

### SDS-PAGE and BN-PAGE

For SDS-PAGE experiments, samples were diluted in SDS-PAGE sample buffer (62.5 mM Tris–HCl, pH 6.8; 10% (v/v) glycerol, 2% (w/v) sodium dodecyl sulfate (SDS), 0.5% bromophenol blue, 2.5% (v/v) β-mercapto-ethanol) and boiled at 95 °C during 5 min. Proteins were then separated at 200 V during 60 min, using 10 or 12% polyacrylamide gel containing 0.35% (V/V) of 2,2,2-trichloroethanol for total protein staining, as described [47]. Briefly, the 2,2,2-trichloroethanol added directly in SDS-PAGE gels interacts with tryptophan in loaded protein and induces UV light-induced fluorescence which can be visualized on a 300 nm transilluminator. Immunolabeling can then be normalized to the total UV light-induced fluorescence (corresponding to the total protein load).

After SDS-PAGE, proteins were transferred to polyvinylidene difluoride (PVDF) membranes. Membranes were blocked for 1 h in TBS-T (50 mM Tris–Cl, pH 7.6; 150 mM NaCl, 0.1% Tween) containing 5% BSA or 5% skimmed milk and incubated with primary antibodies overnight at 4 °C. Protein immunodetection was performed using primary antibodies directed against Src (#2108S, Cell Signaling), NDUFA9 (ab14713, Abcam), UQCRC2 (ab14742, Abcam), COXIV (ab16056, Abcam), ATPB (ab14730, Abcam), VDAC (ab14734, Abcam), TOM20 (sc-17764, Santa Cruz), SOD2 (#13194S, Cell Signaling), cytochrome *c* (ab133504, Abcam), ATP5α (ab14748, Abcam), myc (#2276S, Cell Signaling), α-tubulin (#3763S, Cell Signaling), Drp1 (#8570S, Cell Signaling), FIS1 (#ALX-210-1037-0100, Enzo life sciences), MiD51 (#20164-1-AP, Proteintech), OPA1 (#612607BD, Biosciences), ERp57 (#AF8219, R&D Systems), Smac-Diablo (#15108S, Cell Signaling), V5 (#13202S, Cell signaling) and FLAG (#F1804, Sigma-Aldrich).

### Electron microscopy

*Src*^+/+^ and *Src*^−/−^ MEFs were fixed with 2.5% glutaraldehyde in 0.1 M sodium cacodylate buffer pH 7.4 at 4 °C. Samples were then incubated with 1% osmium tetroxide and 1% potassium ferricyanide in 0.1 M sodium cacodylate buffer for 1 h at 4 °C. After three water washes, samples were dehydrated in a graded ethanol series and embedded in epoxy resin. Ultrathin sections (60–70 nm) were obtained with an Ultrotome V (LKB) ultramicrotome, counterstained with uranyl acetate and lead citrate and viewed with a Tecnai G^2^ (FEI) transmission electron microscope operating at 100 kV. Images were captured with a Veleta (Olympus Soft Imaging System) digital camera at the Imaging facility of the department of biology at the University of Padova (Italy).

The area of individual mitochondria (mitochondrial area, *n* = 131–132) and the perimeter of individual mitochondria (mitochondrial perimeter, *n* = 131–133) were then analyzed using Fiji.

### Cellular respiration

Oxygen consumption was measured using the high-resolution respirometry system Oxygraph-2k Oroboros (Innsbruck, Austria). Cell respiration was measured with 1 × 10^6^ cells mL^−1^ according to volume-specific flux at 37 °C in 2 mL chambers at a stirring rate of 750 rpm. Three different states of endogenous respiration with intact cells were measured: (i) basal respiration representing the endogenous physiological coupled state, (ii) respiration with oligomycin (2 μg mL^−1^) representing the non-coupled resting respiration, and (iii) maximal uncoupled respiration induced by FCCP (0.5 μM steps with 2.5 μM final concentration) providing a measure of the maximal capacity of ETS under conditions of physiological substrate supply in the intact cells.

### Citrate synthase activity

Citrate synthase enzymatic activity was determined with a BioTek Synergy H1 microplate reader (Biotek, Montréal, QC, Canada) at 37 °C by following the reduction of 5,5′-dithiobis (2-nitrobenzoic acid) (ε = 13.6 mL cm^−1^ µmol^−1^) at 412 nm for 8 min. The reaction medium contained 0.1 mmol L^−1^ DTNB, 0.1 mmol L^−1^ Acetyl-CoA and the reaction was started with the addition of 0.13 mmol L^−1^ oxaloacetic acid in 100 mmol L^−1^ imidazole–HCl, pH = 8.

### Statistical analyses

Data are presented as mean ± SEM. Statistical analyses were performed using GraphPad Prism 9. Data were analyzed using Student *t* test, one-way or two-way ANOVA followed by Tukey post hoc test, as appropriate. For two-way ANOVA and post hoc tests, statistical differences (*p* < 0.05) are presented with letters: datapoints with different letters are statistically different (*p* < 0.05). For instance, a datapoint with the letter *a* is statistically different (*p* < 0.05) from datapoints with the letters *b* or *bc*, whereas it is not statistically different (*p* > 0.05) from datapoints with the letters *a* or *ab.*

### Supplementary Information

Below is the link to the electronic supplementary material.

Figure S1. (a) Representative immunoblotting (n=3) of Src and total protein load (TPL) in *Src*^+/+^ and *Src*^−/−^ MEFs. (b) Representative immunoblotting (n=3) of Src and TPL in naive MEFs treated with shRNA as indicated. (c) Representative micrographs (n=3) of MEFs expressing mtGFP and treated with shRNA as indicated. Scale bars: 20 μm. (d, e) Quantitative analysis of mitochondrial morphology showing (d) the distribution of cells among the different mitochondrial phenotypes, (e) the number of mitochondria and the area of individual mitochondria in region of interest (ROI) in MEFs as shown in c. (f) Representative immunoblotting (n=3) of Src and TPL in HeLa cells treated with siRNA as indicated. (g) Quantification of mitochondrial fusion and fission events in live HeLa cells treated with siRNA as indicated and expressing mtGFP (n=3). Cells were imaged during 450 s. See movies 3 and 4. (h) Representative immunoblotting and quantification (n=4-5) of Src, Drp1 and TPL in total cell lysate (TCL), cytosol- (Cyto) and mitochondria-enriched fraction (Mito) obtained from *Src*^+/+^ and *Src*^−/−^ MEFs. Protein levels were normalized by TPL. (i) Representative immunoblotting and quantification (n=3) of OPA1 monomers and oligomers in *Src*^+/+^ and *Src*^−/−^ MEFs treated with the crosslinker BMH. Protein levels were normalized by TPL. (j) Cellular respiration in HeLa cells transfected with scramble siRNA (siCTL) or siRNA targeting Src (siSrc) (n=3). Data are shown as mean ± s.e.m. A.U.: arbitrary unit. Data with different letters are statistically different (p<0.05); *p< 0.05. d, g, h and j were analysed by two-way ANOVA followed by Sidak’s multiple comparison test; e and i were analysed by Student’s t-test (EPS 1366 kb)

Figure S2. (a) Representative immunoblotting (n=3) of Src and V5 in *Src*^+/+^ and *Src*^−/−^ MEFs expressing different constructs as indicated. (b) Representative immunoblotting (n=3) of Src and V5 in HeLa cells over-expressing different constructs as indicated. (c) Representative micrographs (n=3) of wheat germ agglutinin (WGA), V5 and TOM20 labeling in HeLa cells over-expressing pcDNA, Src-V5, mitochondria-targeted mtSrc-V5 and plasma membrane-targeted pmSrc-V5. (d) Quantitative analysis of mitochondrial morphology showing the distribution of cells among the different mitochondrial phenotypes, the number of mitochondria and the area of individual mitochondria in a region of interest (ROI) as shown in g. Scale bars: 20 μm. Data are shown as mean ± s.e.m. Data with different letters are statistically different (p<0.05), according to one-way ANOVA followed by Tukey’s post hoc test (EPS 7220 kb)

Figure S3. Over-expression of Src reduces mitochondrial size in a kinase-dependent manner. (a) Representative micrographs (n=3) of HeLa cells expressing different constructs and labeled as indicated. (b) Quantitative analysis of mitochondrial morphology showing the distribution of cells among the different mitochondrial phenotypes, the number of mitochondria and the area of individual mitochondria in a region of interest (ROI) in HeLa cells as shown in a. Scale bars: 20 μm. Data are shown as mean ± s.e.m. Data with different letters are statistically different (p<0.05), according to one way ANOVA followed by Tukey’s post hoc test (EPS 22504 kb)

Movie 1. Live cell imaging of *Src*^+/+^ MEFs expressing mtGFP. See fig. 2d for quantification of mitochondrial fusion and fission events. The white arrow indicates a representative fusion event and the yellow arrow indicates a representative fission event.

Movie 2. Live cell imaging of *Src*^−/−^ MEFs expressing mtGFP. See fig. 2d for quantification of mitochondrial fusion and fission events. The white arrow indicates a representative fusion event and the yellow arrow indicates a representative fission event

Movie 3. Live cell imaging of HeLa cells treated with siSrc and expressing mtGFP. See fig. S1c for quantification of mitochondrial fusion and fission events. The white arrow indicates a representative fusion event and the yellow arrow indicates a representative fission event

Movie 4. Live cell imaging of HeLa cells treated with siCTL and expressing mtGFP. See fig. S1c for quantification of mitochondrial fusion and fission events. The white arrow indicates a representative fusion event and the yellow arrow indicates a representative fission event

## Data Availability

Complete data or material used in the present study will be available upon reasonable request.
